# Synthesis and Characterization of Novel Inorganic-Organic Hybrid Ru(II) Complexes and Their Application in Selective Hydrogenation

**DOI:** 10.3390/molecules15021028

**Published:** 2010-02-23

**Authors:** Ismail Warad, Zeid Al-Othman, Saud Al-Resayes, Salem S. Al-Deyab, El-Refaie Kenawy

**Affiliations:** 1 Department of Chemistry, King Saud University, P. O. Box 2455, Riyadh 11451, Saudi Arabia; 2 Petrochemical Research Chair, Department of Chemistry, King Saud University, P. O. Box 2455, Riyadh 11451, Saudi Arabia

**Keywords:** Ru(II) complexes, diphosphine, hydrogenation, solid state NMR

## Abstract

Novel Ru(II) complex-based hybrid inorganic-organic materials immobilized via a diamine co-ligand site instead of the conventional diphosphine ligand have been prepared. The complexes were prepared by two different methods: sol-gel and surface modification techniques. The structures of the desired materials were deduced by several available physical measurements like elemental analyses, infrared, FAB-MS and ^1^H-, ^13^C- and ^31^P-NMR spectroscopy. Due to a lack of solubility the structures of xerogel **3** and modified **4** were studied by solid state ^13^C-, ^29^Si- and ^31^P-NMR spectroscopy, infrared spectroscopy and EXAFS. These materials were stable enough to serve as hydrogenation catalysts. Selective hydrogenation of functionalized carbonyls in α,β-unsaturated compounds was successfully carried out under mild conditions in a basic medium using these complexes as catalysts.

## 1. Introduction

It has been shown that complexes containing a "Ru-(P-P)" (P-P = diphosphine) core per ruthenium atom are efficient catalysts in several chemical reactions [1,2,3,4,5]. As a result, several ruthenium complexes containing this motif were synthesized and their properties were studied by spectroscopic and electrochemical techniques [6,7]. Stereo-, regio- and enantioselective ruthenium-catalysis lies at the heart of current developments in pharmaceutical, agrochemical and cognate industries [8,9,10]. Recently ruthenium(II) complexes with diphosphine and diamine ligands were successfully tested as homogenous catalysts in hydrogenation of unsaturated ketones [11,12,13,14,15,16,17,18,19,20]. One of the prime concerns of industry and academia is how to transfer the active homogenous catalysts to the heterogeneous phase [20,21,22,23]. The two major problems in this field have been first that under the conditions commonly required for effective catalysis, ligand exchange reactions lead to leaching of the metal centers from the matrix, in addition to the synthetic difficulties [20,21,22,23]. 

The use of functionalized multidentate ligands with anchoring groups instead of the classical monodentate ones might limit the leaching problem, but few such ligands have been described, probably because of their difficult preparation, specially when the complexes were supported by the phosphine ligands site [20,21,22,23].

Our conceptual methodology was to take a homogeneous system of known catalytic behavior and, by performing suitable modifications, to tether this catalyst on silica supports, covalent anchoring of metal complexes on such surfaces being a topic of considerable interest in catalysis [20,22]. The desired hybrid materials are able to combine the advantages of homogeneous and heterogeneous catalysis: the catalyst becomes easily separable from the reaction products and it can be reused in several runs without an essential loss in the activity due to the leaching problem [23,24,25,26].

In this work we used a bidentate diphosphine ligand and diamine co-ligand with Si(OMe)_3 _anchoring groups; the presence of this group enables the immobilization of the ruthenium(II) complexes through simple a sol-gel process using HSi(OEt)_3_ as cross-linker. Another approach to achieve stable materials is to covalently bind the ligands, *via* the methoxy groups, to the well known silica surface instead of a sol-gel material through surface modification. 

## 2. Results and Discussion

### 2.1. Synthesis of ruthenium(II) complexes [1-4]

**Scheme 1:**
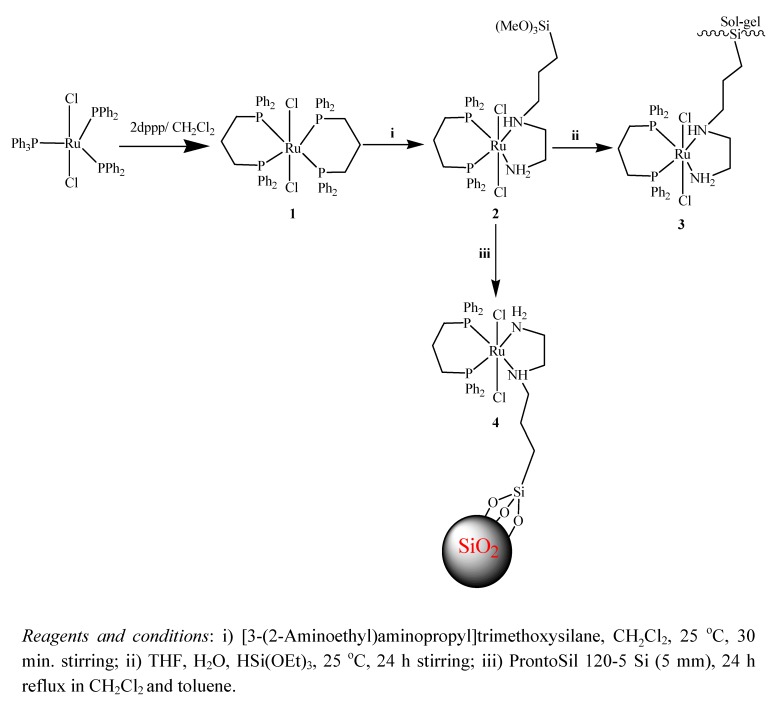
The synthetic route used in the preparation of the hydrogenation catalyst **2**, the sol-gel xerogel **3**, and the modified **4**.

Our approach to the preparation of the stationary phase catalysis system is shown in Scheme 1. The precursor compound *trans*-Cl_2_Ru(dppp)_2_ (**1**) was obtained by a substitution reaction starting from Cl_2_Ru(PPh_3_)_3_ and dppp in dichloromethane [4,14]. Complex **2** was prepared by treating **1** with equimolar amounts of [3-(2-aminoethyl)-aminopropyl]trimethoxysilane in dichloromethane; in order to confirm this ligand exchange the reaction was monitored by ^31^P-NMR. One of the dppp ligands was replaced by one of the diamine ligands [4,14], giving a yellow product which were collected in excellent yield. It is soluble in chlorinated solvents such as chloroform or dichloromethane and non-soluble in polar or non-polar solvents like water, methanol or diethyl ether and *n*-hexane. By condensation of **2**, which contains a trimethoxysilane functional group, with 10 equivalents of HSi(OEt)_3_ in THF/water under sol-gel conditions the xerogel **3** was prepared in good yield. In another route **2** was modified using ProntoSIL 120-5 Si (5µm) as support in toluene to produce **4**. The yield of the immobilized complexes **3** and **4** was very good, but the hybrid materials were not soluble in any solvent.

### 2.2. NMR and IR spectroscopic investigations

The structure of complexes **1** and **2** described herein has been deduced from elemental analyses, infrared, FAB-MS and ^1^H-, ^13^C-, H, H-COSY and ^31^P-NMR spectroscopy. Due to lack of solubility the structures of **3** and **4** were determined by solid state ^13^C-, ^29^Si- and ^31^P-NMR spectroscopy, infrared spectroscopy, and EXAFS. 

**Figure 1:**
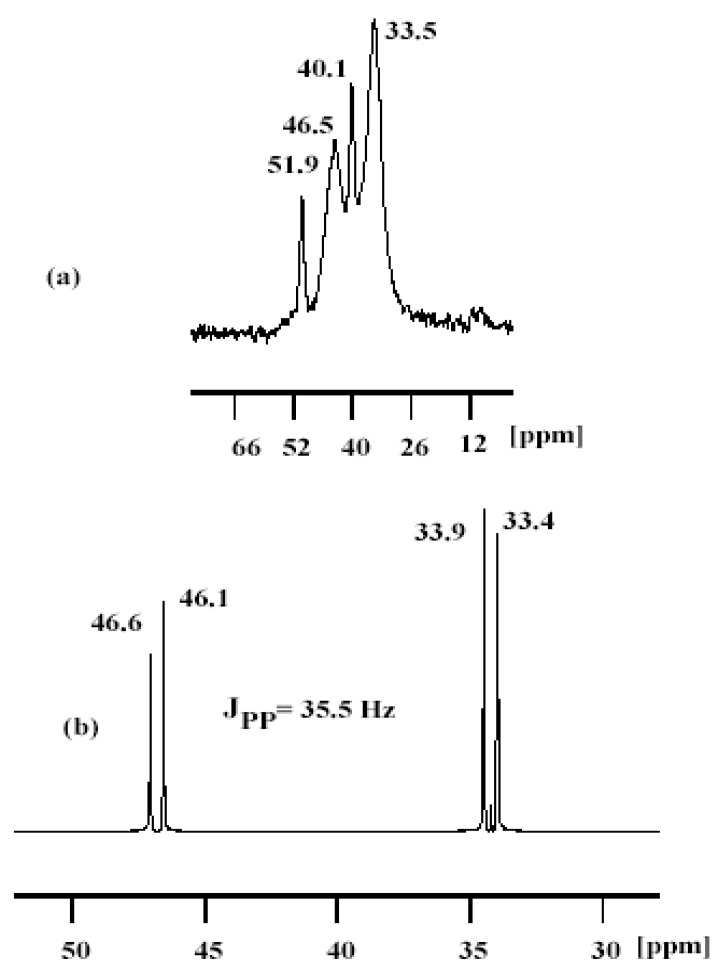
(a) ^31^P-CP/MAS-NMR spectrum of the modified **4**, (b) ^31^P{^1^H} of complex **2** in CD_2_Cl_2. _

Confirmation of the formation of **3** and **4 **was obtained by NMR. Liquid ^31^P-NMR and solid state ^31^P-CP/MAS-NMR measurements for both **2** and the interphased Ru(II) complexes in the hybrid xerogel **3 **and surface modified **4 **support the formation of the expected structural complexes whose formulae are shown in Scheme 1. The use on an asymmetric diamine caused loss of the *C_2_* axis resulting in a splitting of the ^31^P resonance into AX patterns, as illustrated in Figure 1. The phosphorous chemical shifts and the ^31^P-^31^P coupling constants (*J*_pp_ = 35.5 Hz) suggested that the dppp ligand was positioned *trans* to the diamine, with *trans* dichloro atoms, to form the kinetically favored *trans*-Cl_2_Ru(II) isomer [14,27], while the thermodynamic favored *cis*-Cl_2_Ru(II) isomer [28,29] was not detected according to the ^31^P-CP/MAS-NMR data. 

The ^31^P-MAS-NMR spectra of the xerogel **3** and modified material **4** are presented together to elucidate the structural nature and integrity of the ruthenium(II) supported complexes. The ^31^P-MAS NMR spectrum of the modified **4** complex is presented together with the liquid ^31^P-NMR spectrum of **2** (Figure 1). In the case of **4 **(Figure 1a) relatively broad resonances centered at 33.5, 40.1, 46.5, 51.9 ppm were observed. Fortunately, even in the presence of the line broadening the chemical shifts of **4** can be assigned, which support the formation of the expected *trans* Cl_2_Ru(II) isomer in the solid state as in liquid state (Figure 1b) and the Ru–P bond seemed to not be affected during the grafting; free ligand was not detected (δ_p_ = - 21.8 ppm) and other chemical shifts corresponding to coordination of phosphine to another species are also not recorded. 

**Figure 2:**
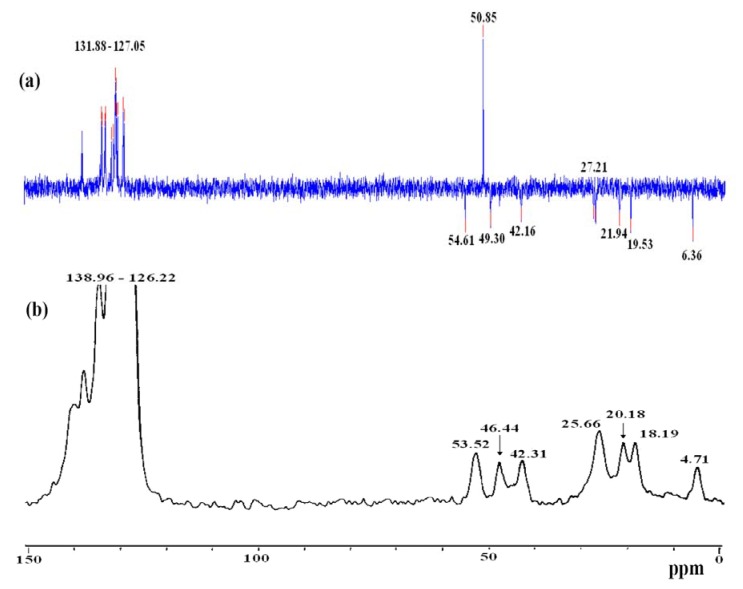
(a) Dept 135^ 13^C-NMR of **2** in CD_2_Cl_2_ (b) ^13^C-CP/MAS-NMR spectra of the xerogel **3**_._

Examination of the ^13^C-CP-MAS-NMR spectrum of the modified solids along with the solution phase spectrum of the corresponding molecular precursor led to the conclusion that the organic fragments in **3 **and **4 **remained intact during the grafting and subsequent workup without measurable decomposition (Figure 2). The absence of the methoxy groups at δ_C_ = 50.8 ppm after the immobilization processes in **3** and **4**, compared by **2**, were the major differences noted between spectra, which supported the immobilization of the desired hybrid Ru(II) complexes. The total disappearance of (CH_3_O)_3_Si groups in **3** (Figure 2b), compared by **2** (Figure 2b), provides good confirmation of a sol-gel process gone to full completion.

**Figure 3:**
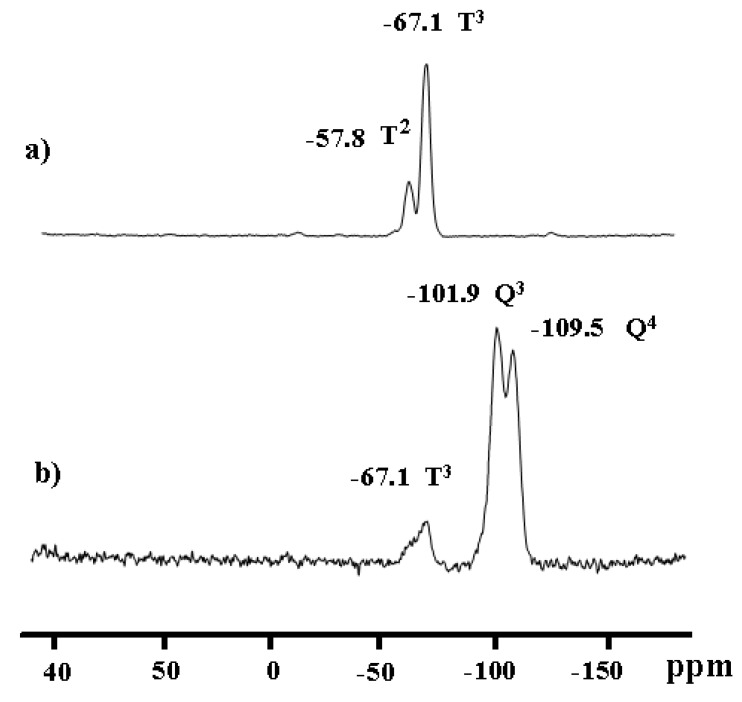
a) CP-MAS-^29^Si-NMR of xerogel **3 **sol-gel functionalized complex; and **b**) surface modified material **4**.

Solid-state ^29^Si-NMR provided further information about the silicon environment and the degree of functionalization [30]. In all cases, the organometallic/organic fragment of the precursor molecule was covalently grafted onto the solid, and the precursors were, in general, attached to the surface of the polysiloxane (as in **3**) or mesoporous oxide by multiple siloxane bridges (as in **4**) as evidenced in Figure 3. The presence of T^m^ sites in case of xerogel **3** (with m = 2 and 3) in the spectral region of T^2^ at δ_Si_ = -57.8 ppm and T^3^ at δ_Si_ = - 67.1 ppm as expected, and no peaks belonging to Q silicon sites were detected, as in Figure 3a. Peaks assignable to Q^3^ at δ_Si_ = -101.9 ppm and Q^4^ at δ_Si_ = -109.5 ppm silicon sites of the silica framework in addition to T^3^ at δ_Si_ = - 67.1 ppm were discernible in modified **4**, as seen in Figure 3b.

The IR spectra of the complexes **2-4 **in particular show four sets of characteristic absorptions in the ranges 3,336–3,319, 3,268–3,215, 3,178–3,165, and 275–254 cm^-1^, which can be assigned to NH_2_, amine-CH, phosphine-CH and RuCl stretching vibrations, respectively.

### 2.3. EXAFS measurement of xerogel ***3***

**Figure 4:**
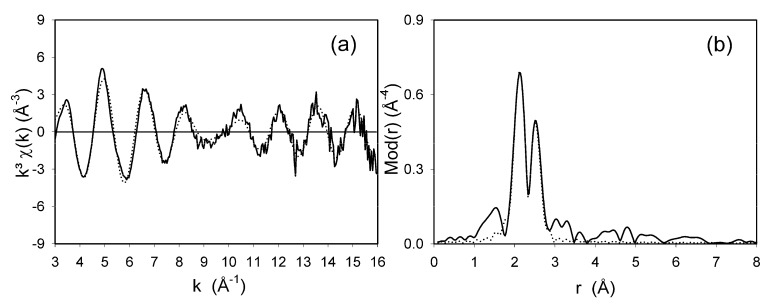
Experimental (solid line) and calculated (dotted line) EXAFS functions (a) and their Fourier transforms (b) for xerogel **3** measured at Ru K-edge.

One of the most powerful methods to obtain interatomic distances of amorphous materials is extended X-ray absorption fine structure spectroscopy (EXAFS). The xerogel **3** was chosen as an example to determine the bond lengths between the metal center and the coordinating atoms of the ligand. The k^3^ weighted EXAFS function of xerogel **3** can be described best by six different atom shells. The first intensive peak in the corresponding Fourier transform (Figure 4a) is mainly due to the nitrogen atoms. Chlorine and phosphorus atoms were found in the case of the most intense peak. For the most intense peak of the Fourier Transform, two equivalent phosphorus, two nitrogen atoms and two chlorine atoms with Ru-P, Ru-N and Ru-Cl bond distances of 2.26, 2.19 and 2.41 Å, respectively, were found (Figure 4b and Table 1). These results reveal a good agreement between the experimental and the calculated functions. 

**Table 1:** EXAFS determined structural parameters of xerogel 3.

A-Bs^a^	N^b^	R ^c ^[Å]	σ ^d ^[Å]	ΔE_0_^e ^[eV]	*k*-range [Å^-1^]	Fit-Index
Ru – N	2	2.19 ± 0.02	0.050 ± 0.005	22.72	3.0 - 16.0	32.24
Ru – P	2	2.26 ± 0.02	0.055 ± 0.006			
Ru – Cl	2	2.41 ± 0.03	0.063 ± 0.007			

^a^ absorber (A) – backscatterers (Bs),^b^ coordination number N, ^c^interatomic distance r, ^d^Debye-Waller factor σ with its calculated deviation and ^e^ shift of the threshold energy ΔE_0_.

### 2.4. Catalytic activity of complexes ***2-4*** in the hydrogenation of α,ß-unsaturated carbonyl compounds.

**Scheme 2:**
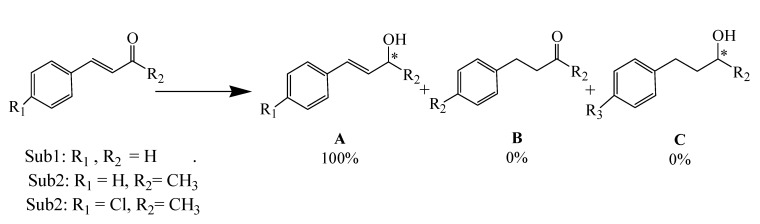
Hydrogenation of α,ß-unsaturated carbonyl compounds possibilities.

The catalytic activity of the desired hybrid ruthenium(II) materials **2-4**, was carried out using α,ß-unsaturated carbonyl compounds in 2-propanol as solvent, and the chosen α,ß-unsaturated carbonyl compounds allowed us to study the selectivity of the catalysts. Three different outcomes of the hydrogenation process are to be expected (Scheme 2). 

The Ru(II) complexes have been tested as catalyst precursors for hydrogenation under mild conditions: 2 bar hydrogen pressure, six equivalents of KOH, 35 ^o^C and with a molar substrate to catalyst (S/C) ratio of 1,000:1. The hydrogenation results are summarized in Table 2.

**Table 2:** Hydrogenation results of α,β-unsaturated carbonyl compounds. ^a^

	Substrate	Complex	Conversion (%)^b^	C=O Selectivity (%)^b^	TOF^c^
1	Sub1	1	0	0	0
2	Sub1	2	>99	>99	1220
3	Sub2	2	>99	>99	1205
4	Sub3	2	>99	>99	1190
5	Sub1	2	0	0	0
6	Sub1	3^d^	98	97	113
7	Sub2	3^d^	95	96	120
8	Sub3	3^d^	93	95	108
9	Sub1	4^d^	97	94	128
10	Sub2	4^d^	95	95	102
11	Sub3	4^d^	96	94	110

^a^ Two model substrate were used, reactions were conducted at 35^o^C, (S/C=1,000) in 50 mL of 2-propanol, P_H2_ =2 bar, [Ru:KOH:Sub] [1:6:1,000]. ^b^ Yield and selectivity were determined by GC. ^c^ TOF: turnover frequency (mol_sub_ mol^-1^_cat_ h^-1^). ^c^ the same conditions of ^a^ but the reactions conversation was determined after 12 h.

As expected RuCl_2_(dppp)_2_** 1** was completely inactive in the hydrogenation of unsaturated ketones under the above reaction conditions (Table 2, trial 1), which was traced back to the absence of the diamine. All the trials of precursor **2** showed more than 99% activity and hydrogenation selectivity towards the C=O group in the presence of a C=C group with high TOF, (Table 2, trials 2-4). In the absence of KOH co-catalyst and under identical conditions the active precursor **2** became totally inactive (Table 2, trials 2 and 5), which supports the idea that the role of the co-catalyst is to activate the catalyst by forming the ruthenium(II) hydride intermediate [10,11,12,13,14,15,16,17,18,19,20].

All of the other ruthenium(II) precursors **3** and **4** display high conversion ratios and selectivity ~ 95% in the hydrogenation of the target substrates (Table 2, trials 6-11). Expected constant decrease in the turnover frequencies (TOFs) and the conversions were observed by comparing the homogenous **2** with the heterogeneous **3** and **4** precursors.

## 3. Experimental

### 3.1. General remarks, materials, and instrumentation

All reactions were carried out in an inert atmosphere (argon) by using standard high vacuum and Schlenk-line techniques, unless otherwise noted. Prior to use CH_2_Cl_2_, *n*-hexane, and Et_2_O were distilled from CaH_2_, LiAlH_4_, and from sodium/benzophenone, respectively. 1,3−Bis(diphenyl-phosphino)propane (dppp) was prepared according to literature methods [14]. 3-(2-Aminoethyl)amino-propyl]trimethoxysilane was purchased from Acros and had to be purified by distillation and recrystallization, Elemental analyses were carried out on an Elementar Vario EL analyzer. High-resolution liquid ^1^H-, ^13^C{^1^H}-, DEPT 135, and ^31^P{^1^H}-NMR spectra were recorded on a Bruker DRX 250 spectrometer at 298 K. Frequencies are as follows: ^1^H-NMR: 250.12 MHz, ^13^C{^1^H}-NMR: 62.9 MHz, and ^31^P{^1^H}-NMR 101.25 MHz. Chemical shifts in the ^1^H- and ^13^C{^1^H- NMR spectra were measured relative to partially deuterated solvent peaks which are reported relative to TMS. ^31^P chemical shifts were measured relative to 85% H_3_PO_4_. CP/MAS solid-state NMR spectra were recorded on Bruker DSX 200 (4.7 T) and Bruker ASX 300 (7.05 T) multinuclear spectrometers equipped with wide-bore magnets. Magic angel spinning was applied at 4 kHz (^29^Si) and 10 kHz (^13^C, ^31^P) using (4 mm ZrO_2_ rotors). Frequencies and standards: ^31^P, 81.961 MHz (4.7 T), 121.442 MHz (7.05 T) [85% H3PO4, NH4H2PO4 (d = 0.8) as second standard]; ^13^C, 50.228 MHz (4.7 T), 75.432 MHz (7.05 T) [TMS, carbonyl resonance of glycine (δ = 176.05) as second standard]; ^29^Si, 39.73 MHz (4.7 T), 59.595 MHz (7.05 T, (Q8M8 as second standard). All samples were prepared with exclusion of molecular oxygen. IR data were obtained on a Bruker IFS 48 FT-IR spectrometer. Mass spectra: EI-MS, Finnigan TSQ70 (200 ^o^C) and FAB-MS, Finnigan 711A (8 kV), modified by AMD and reported as mass/charge *(m/z). *The analyses of the hydrogenation experiments were performed on a GC 6000 Vega Gas 2 (Carlo Erba Instrument) with a FID and capillary column PS 255 [10 m, carrier gas, He (40 kPa), integrator 3390 A (Hewlett Packard)]. The EXAFS measurements were performed at the ruthenium K–edge (22118 eV) at the beam line X1.1 of the Hamburger Synchrotronstrahlungslabor (HASYLAB) at DESY Hamburg, under ambient conditions, energy 4.5 GeV, and initial beam current 120 mA. For harmonic rejection, the second crystal of the Si(311) double crystal monochromator was tilted to 30 %. Data were collected in transmission mode with the ion chambers flushed with argon. The energy was calibrated with a ruthenium metal foil of 20 μm thickness. The samples were prepared of a mixture of the samples and polyethylene.

### 3.2. General procedure for the preparation of the complex ***2***

3-(2-Aminoethyl)aminopropyl]trimethoxysilane (0.035 mL, 0.55 mmol, 10% excess) was dissolved in dichloromethane (10 mL) and the solution was added dropwise to a stirred solution of **1** (500 mg, 0.50 mmol) in dichloromethane (10 mL) within 5 min. The mixture was stirred for ca. 2 h at room temperature while the color changed from brown to yellow. After removal of any turbidity by filtration (P4), the volume of the solution was concentrated to about 5 mL under reduced pressure. Addition of diethyl ether (40 mL) caused precipitation of a solid, which was filtered (P4). After recrystallization from dichloromethane/*n*-hexane, complex **2** was obtained in analytically pure form. Yield 312 mg (93%) of a yellow powder, m.p. 340 ^o^C (dec.); ^1^H-NMR (CDCl_3_): δ (ppm) 0.11 (m, 2H, CH_2_Si), 0.60 (br, 2H, PCH_2_C*H_2_*), 1.43 (br, 4H, PC*H_2_*CH_2_), 2.07 (m, 2H, C*H_2_*CH_2_Si), 2.45 (m, 4H, N*CH_2_CH_2_*N), 2.93 (m, 5H, NH_2_, NH, NHCH_2_), 3.49 (s, 9H, OCH_3_) 7.10- 7.60 (m, 20H, C_6_H_5_); ^31^P{^1^H}-NMR (CDCl_3_): δ (ppm) 33.50, 46.35, dd, J_pp_ =35.5 Hz; ^13^C{^1^H}-NMR (CDCl_3_): δ (ppm) 6.32 (s, 1C, CH_2_Si), 19.55 (s, 1C, PCH_2_*C*H_2_), 21.92 (m, 1C, *C*H_2_CH_2_Si), 27.83 (m, 2C, PCH_2_), 42.84 (s, 1C, NH_2_CH_2_), 49.34 (s, 1C, NH*C*H_2_CH_2_CH_2_) 50.84 (s, 3C, OCH_3_), 54.62 (s, 1C, NH_2_*C*H_2_CH_2_NH), 126.23-136.82 (m, 24C, C_6_H_5_); FAB – MS; *(m/z)*: 806.2 (M^+^); Anal. Calc. C, 52.11; H, 6.00; Cl, 8.79; N, 3.47 for C_35_H_48_Cl_2_N_2_O_3_P_2_RuSi: Found C, 52.04; H, 6.22; Cl, 8.70; N, 3.35%.

### 3.3. General procedure for sol–gel processing of xerogel ***3***

Compound **2** (300 mg, 0.235 mmol) and HSi(OMe)_3_ (10 equivalents) in methanol (10 mL) were mixed together. The sol–gel took place when a THF/water mixture (4 mL, 1:1 v/v) was added to the solution. After 24 h stirring at room temperature, the precipitated gel was washed with toluene and diethyl ether (50 mL of each), and petroleum ether (40 mL). Finally the xerogel was ground and dried under vacuum for 24 h to afford after workup 500 mg of **3** as a pale yellow powder. ^31^P-CP/MAS-NMR: δ = 33.50, 46.35. dd, J_pp_ =35.5 Hz; ^13^C-CP/MAS NMR: δ (ppm) 4.71 (br, 1C, CH_2_Si), 18.19 (br, 1C, PCH_2_*C*H_2_), 20.18 (m, 1C, *C*H_2_CH_2_Si), 25.66 (m, 2C, PCH_2_), 42.31 (br, 1C, NH_2_CH_2_), 46.44 (s, 1C, NH*C*H_2_CH_2_CH_2_), 53.52 (s, 1C, NH_2_*C*H_2_CH_2_NH), 126.22-138.96 (m, 24C, C_6_H_5_); ^29^Si CP/MAS NMR: δ = –67.1 ppm (T^3^), –57.2 ppm (T^2^).

### 3.4. General procedure for surface modifiedmaterial ***4***

Compound **2 **(0.300 mg, 0.235 mmol) dissolved in CH_2_Cl_2_ (50 mL) was added dropwise to a suspension of ProntoSil 120-5 Si (5 mm) (0.5 g) in dry toluene (50 mL) and stirred at 25 °C for 2 h to allow the diffusion of the molecular precursor into the pore channels. The reaction mixture was then refluxed for 24 h. After filtration, the unreacted ruthenium precursor was removed by thoroughly washing the solid twice with toluene then CH_2_Cl_2_ (25 mL each). Finally, the resulting solid was dried *in vacuo* (~ 0.40 atm) at 30 °C to afford 620 mg of **4** as a pale white powder. ^31^P-CP/MAS-NMR: δ = 33.80, 46.42. dd, J_pp_ =35.5 Hz; ^13^C CP/MAS-NMR: δ (ppm) 4.82 (br, 1C, CH_2_Si), 18.39 (br, 1C, PCH_2_*C*H_2_), 21.08 (m, 1C, *C*H_2_CH_2_Si), 26.34 (m, 2C, PCH_2_), 42.81 (br, 1C, NH_2_CH_2_), 46.76 (s, 1C, NH*C*H_2_CH_2_CH_2_), 53.82 (s, 1C, NH_2_*C*H_2_CH_2_NH), 126.34-138.77 (m, 24C, C_6_H_5_); ^29^Si-CP/MAS- NMR: δ = –67.1 (T^3^) and –57.2 (T^2^), -101.9 ppm (Q^3^) and -109.5 ppm (Q^4^).

### 3.5. General procedure for the catalysis studies

The respective matrix of diamine(dppp)ruthenium(II) supported complexes, (100 mg, 6% Ru) was placed in a 250 mL pressure Schlenk tube, a calculated amount of KOH (0.06 mmol, 10 equivalents) was added as co-catalyst, then *trans*-4-phenyl-3-butene-2-one (60 mmol, 1000 equivalents) was added. The solid mixture was magnetically stirred and warmed during the evacuation process to remove oxygen and water. Subsequently the Schlenk tube was filled with argon and 2-propanol (80 mL). The mixture was vigorously stirred, degassed by two freeze-thaw cycles, and then sonicated for 30 min (this is important to increase the homogeneity of the mixture). Finally the reaction mixture was pressurized with H_2 _(2 bar) after flushing with H_2_ three times. The reaction mixture was vigorously and magnetically stirred at 35 °C for 1−12 h. During the hydrogenation process samples were taken from the reaction mixture to check the course of the reaction. The samples were inserted by a special glass syringe into a gas chromatograph and the reaction products compared with authentic samples.

## 4. Conclusions

Four new complexes were prepared by a novel, fast, easy, one step ligand exchange technique and characterized. The presence of T-silyl functions on the diamine co-ligand backbone enable the hybridization of these complexes in order to support them on a polysiloxane matrix through sol-gel or surface modification processes. When the polymeric complexes presented in this investigation were tested as catalysts for the hydrogenation of unsaturated ketones, they showed high degree of stability and activity as well as an excellent degree of carbonyl hydrogenation selectivity under mild conditions.
